# Supplementary material for: Chromothripsis during telomere crisis is independent of NHEJ, and consistent with a replicative origin
Source: Genome Res. 2019 May;29(5):737–49. doi: 10.1101/gr.240705.118 (PMC6499312; doi:10.1101/gr.240705.118)
Supplement: Supplemental Material [file supp_gr.240705.118_Supplemental_file_1.zip › contigs/annotated_contigs/DB50/contig.2.DB50_length_626_mean_cov_12.3514376997.docx]

**DB50_length_626_mean_cov_12.3514376997**

```
GTCATGGTAGAAGGTTGGTCTA|CATCCACCTCACCTTTGTGATCTTCTGAGGGAGCCGCAGCCAGTGTGTAAGGGAAGCAGGGGTAGA
                       >chr16:81497684-81497958 + E=4e-150
GACAGTAGCTTTGTCATTAGTCTCCGCATTTCACAGGTGAGAAAAGGAGTCTCCCAGGGGAAGTGGCGTGGGCACAGTGAGAGGTGGCT

GGCTAAGCCAGAGCTCTTCCTAGTGCCAGCACCTTCCTAGAATTAGCTGTGAGACTGAAGAGAGGAAAGATGACATCTGGTCACAGGCT

CCACATCTGGTCCGATGGGGCAGGTGCCT|T|AGTTTATACTAGCCTTTCCCACGTCAACTTGAAAACACTGCCCTTTGTTATGCAAAA
                              >chr16:81396784-81397115 + E=2e-187
ATAATTGTATTGAACCAGAGGTAACAGTTCATTTTCTTGTTGTGAGCTATTATGGGTTAAAAAGTGAGGATTTTCTGATGAACTCACCA

TGTATTTCTTAAGTTTGTTTTGAGAAGTGGCTTTGGCCTCTCTGGCTGTTGTGCTGGAAGAGTGGGCTGTTAGGGAAGCCAGAAATCTT

TAAGAAGCATTACCGGAGAAATGGTTTTGAACGTGGTACTTCCATGCTGGAACACTGGGAAAAAAGATTTCTCTAATTTTTAAAAATAG

TTTCAA
```
